# Female blind baseball players against gender discrimination: the “red diamonds” experience

**DOI:** 10.3389/fspor.2024.1362664

**Published:** 2024-04-25

**Authors:** Giuditta Carretti, Pippo Russo, Mirko Manetti, Mirca Marini

**Affiliations:** ^1^ Section of Anatomy and Histology, Department of Experimental and Clinical Medicine, University of Florence, Florence, Italy; ^2^ Department of Political and Social Sciences, University of Florence, Florence, Italy

**Keywords:** visual disability, adapted baseball, female sport, gender discrimination, dual embodiment, empowerment, social integration, body image

## Abstract

**Background:**

Sport has the well-known power of improving body awareness, self-esteem, and social interaction, thus promoting quality of life and psychophysical wellbeing. Specifically referring to adapted disciplines, habitual practice often becomes an effective integration and self-efficacy booster. Among disabilities, visual impairment deeply alters body image perception, autonomy, and environmental/social interaction heavily reducing sport or leisure involvement opportunities. In particular, visually impaired women represent one of the most vulnerable categories to gender and disability discrimination. Moreover, even when congenitally sightless, they perceive social pressure of mainstream beauty ideals, mostly spread by media, comparable to their sighted peers. On these premises and the previously demonstrated psychophysical benefits of Italian blind baseball practice on this target population, the present study aimed to deepen the social and educative potentialities of such adapted sport applying a more sociological research approach.

**Methods:**

The “red diamonds” event, namely, the first ever female blind baseball match, was the setting for the administration of our structured online survey. In detail, our survey comprised different evaluation tools such as the 18-item Psychological Well-Being Scale, the 12-item Short Form questionnaire, the Dresden Body Image questionnaire, the Rosenberg Self-Esteem Scale, and sociological model designed questions. Quality of life, psychological wellbeing, self-esteem, body image, and perceived female sport psychological violence were investigated in the whole women sample (*n* = 33) voluntarily adhering to the game.

**Results:**

Survey results revealed no statistically significant differences between visually impaired players (*n* = 13; mean age: 32.84 ± 12.05 years) and sighted on-field subjects (i.e., coaches, assistants, and referees; *n* = 20; mean age: 47.15 ± 12.31 years) in almost all the inquired variables, thus remarking the social and functional benefits of adapted sport through the “dual embodiment” and empowerment phenomenon.

**Conclusions:**

Given that the event was inspired by and performed on the World Day against women violence, our study deepened not only the topic of disability discrimination but also the currently alarming gender-related one. In such a context, the present research might provide interesting cues for further investigations on disability and gender disparities in sports, hence spreading interest in this under-investigated field. In perspective, the “red diamonds” experience could also contribute to inspiring and progressively developing educative tools against any kind of discrimination by promoting integration and social growth through regular sports practice.

## 1 Introduction

The literature has widely acknowledged physical activity as an essential component of a healthy lifestyle and a determinant of perceived quality of life and psychophysical wellbeing. Despite such growing evidence and the current health recommendations, most all-aged individuals do not meet the established daily movement guidelines, especially those affected by disability (1–3).

Specifically, it is well-known that visual impairment deeply impacts psychological, social, and physical functionality, thus hindering participation in daily-life, leisure, and sport activities (4). According to several studies, visually impaired people perceive and experience prejudices, barriers, accessibility issues, and restrictions in participation in a regular and structured physical activity throughout their lifespan (5–8). Regardless of the cause, type, and severity of visual impairment, the lack of sight impedes to effectively interact with the surrounding environment and other individuals, hence delaying or altering body awareness acquisition (9–11). A recent investigation also highlighted the influence of gender on physical activity and regular engagement of people with disability, detecting lower levels in the female component (12, 13). Sport practice, recreational or competitive, offers an enjoyable and healthy psychophysical and socioemotional growth opportunity to visually impaired people while counteracting disability-related onset of physical and mental disorders (14, 15). Although research addressing these topics is still scarce and almost no exercise guidelines tailored to this target are available, it has been demonstrated that regular physical activity practice plays a key role not only in the structuring of sensorimotor skills but also in the development of personality, autonomy, body awareness, and self-esteem. Visual disability–affected people often perceive a loss of control and reduced self-mastery and abilities in performing daily-life activities and social interaction. Vision loss substantially impacts the overall functionality and the consequent autonomy of these individuals, thus negatively affecting their perceived quality of life and wellbeing (16, 17). Although such parameters have been widely investigated and described as multidimensional constructs resulting from the balance between psychophysical and social challenges and subjective available resources/skills, they still remain not peculiarly defined in visually impaired people (18). The literature repeatedly highlighted the difficulty in assessing the health-related quality of life in this target population though simultaneously acknowledging the key role played on it by physical activity and sport practice (9, 19). Indeed, constant physical exercise engagement enhances cardiorespiratory functionality, postural control, coordination, balance, and social/environmental interactions with consequent improvement in sensorimotor efficiency, autonomy, and the overall health status (4). Moreover, given the multiple values, primarily fair play, effort, discipline, respect, challenge, teamwork, and membership underlying sport engagement, a regular and structured practice may help counteracting the aforementioned disability-related deficits while promoting self-efficacy, social integration, and body confidence (20).

Modern society is strongly based on unreachable esthetic and performative models that can easily undermine self-esteem and body acceptance, especially in particular sensitive age, gender, or health-condition groups (21–23). Even though visual experience has a significant impact on how we interiorize our own body template, recent studies highlighted that, when compared to sighted peers, visually impaired individuals show similar beauty ideals awareness and media pressure perception. Among them, women feel higher conforming and social comparison pressure, hence clearly demonstrating that vulnerability to sociocultural standards of attractiveness is sight independent (24, 25). Media and fashion industries are growingly spreading pro-thin and anti-fat ideals, thus contributing to increased body dissatisfaction, and eating disorders, especially among females. The literature specifically investigating body image and satisfaction in sight-impaired individuals is still scarce. Furthermore, most published studies were limited to small samples and reported heterogeneous results obtained through evaluative tools not yet peculiarly validated for this target population. Despite these criticalities, research addressing beauty standards in congenitally blind women suggested that thin ideal internalization can occur even without any body shape visual exposure, automatically developing a pro-thinness attitude, mostly fueled by the media, comparable to sighted peers (26, 27). Body image is a complex multidimensional construct comprising perceptual, socio-educational, and affective factors. Individuals with disabilities often experience their condition as a social stigma, thus perceiving discrimination to negatively impact and alter their self-concept. Noteworthy, physical appearance and body efficiency involve not only the self-perception of the impaired persons but also the reaction of other individuals to their diversity (28, 29). In the case of vision loss subjects, particularly young women shape this concept on externally provided verbal feedback, tactile body exploration, and perceived feelings/emotions during physical and social interaction (30, 31).

Since sport and physical activity involve all these aspects, urging participants to develop healthy and fair comparisons and to overcome limits, they undoubtedly represent effective tools/facilitators to promote self-confidence, emotional intelligence, inclusiveness, and tolerance beyond any kind of diversity or standard (32–34). From a sociological perspective, Paralympic sports aim to reduce the gap between able-bodied and disability-affected people, therefore representing an integration-oriented phenomenon (35–37). Noteworthy, to realize a real integration, it is crucial to approach/apply such a concept as a two-sided process based on reciprocity as the main interaction driver. In this perspective, both able-bodied and disabled persons are actors who reciprocally integrate each other (38, 39). Concretely, each part must learn to think, act, and perform in a way like the other, and, in particular, able-bodied subjects need to understand the feelings of disability-affected ones, while the latter need to be able to perform under psychophysical and competitive stress as it typically occurs in sport frames (40). Such reciprocal understanding/exchange is a sort of pact characterized by a mutual help dimension aimed to set up good situational cooperation. This reciprocal attitude can be framed as a “dual embodiment” affair meaning such term as a process by which the body becomes a tool to gain experiences; adapt to social and ecological environments; interiorize; and share sensations, socialize individuality, and explore ways of socialization (41–43). Therefore, the body simultaneously represents a tool of action and knowledge, and embodiment is the process by which the knowledge mechanism is performed.

Among disability-adapted sports, the Italian blind baseball (BXC) can be considered a unique model of the aforementioned dynamics given that, as already detailed in our previous study, it officially provides for age, gender, and disability severity–mixed team, contemporarily involving sighted and sight-impaired subjects. Recently, on the World Day against gender violence, the first ever BXC match exclusively played, coached, and refereed by women was performed in Sardinia, Italy. The event, named “red diamonds,” thus recalling the baseball field shape and the color of the symbol against women violence, contributed to paving the way for a more conscious, attentive, and respectful approach to female sport and disabled women integration opportunities (44).

In such a context, the present study aimed to investigate the perceived quality of life, psychological wellbeing, self-esteem, and body image of all the female subjects involved in the match, featuring visually impaired BXC athletes, sighted coaches, field assistants, and referees. Though the BXC regulation provides for and promotes gender-mixed teams, the female subjects, both sighted and sight-impaired, are still underrepresented in this discipline. Therefore, the enrolled women, who voluntarily adhered to the match and to the study, represented almost the entire Italian female component regularly engaged and officially signed up in this adapted competitive sport. Given the uniqueness of the “red diamonds” event and the increasing promotion of this sport carried out by the Italian Blind Baseball Association/League (AIBXC/LIBCI) among women, the match offered the unique opportunity to investigate and deepen the aforementioned variables in female competitive BXC regular practitioners. In addition, involvement in this study of all the provided game roles (i.e., sighted assistants, coaches, referees, and visually impaired athletes) allowed us to explore such variables from different perspectives, contemporarily detecting possible sight- and disability-influenced differences. To collect reliable data, validated qualitative scales and questionnaires were administered to each participant. Moreover, given the intrinsic sociological implications and potentialities of sport, gender, and disability disparities perceived in sport practice, management and media portrayal were also explored through a multiperspective approach. In detail, one of the online survey sections was imprinted on three macro focuses investigating the reason underlying sport practice and “red diamonds” participation, the relationship with corporeal dimension framed to sport field, and the perceived prejudices and psychological violence in female sport, respectively. Such sections were purpose-designed following a question structure typically applied in sociological studies.

## 2 Materials and methods

### 2.1 Participants

The study group consisted of 33 women of whom 29 regularly signed up to one of the BXC teams officially affiliated to the Italian Blind Baseball Association/League (AIBXC/LIBCI) and 4 female referees licensed and acknowledged by the BXC authorities. In detail, the whole women sample was composed of 13 visually impaired competitive athletes and 20 sighted subjects, namely, 2 coaches, 12 field assistants, 2 second-base catchers, and 4 referees. All the participants voluntarily adhered to the “red diamonds” BXC match and, in such a context, they deliberately gave their consent to take part in the present research and anonymously compiled a self-administered online questionnaire. Given that the “red diamonds” event and the study design were concurrently conceived aiming to involve and investigate the whole Italian female component of BXC regular practitioners, both sighted and visually impaired, no inclusion/exclusion criteria were applied to participants. AIBXC and LIBCI detailed and promoted the adhesion to the match, as well as to this study, through their official communication channels and the direct link with the staff of each regularly affiliated competitive team. Study procedures were carried out following the rules of the 1975 Declaration of Helsinki, revised in 2013; this anonymous online survey did not require an ethics committee approval.

### 2.2 Instrument and procedure

The tool employed to collect data consisted of a self-administered online questionnaire purposely designed through the Google Forms platform and distributed to participants in the form of a direct access link shared using the official communication channels of the event. Concerning the modality of survey administration and answering, visually impaired athletes took advantage of specific assistive technologies such as speech synthesis or video magnifiers. All the completely anonymous and confidential responses were recorded in the Google Forms database (45).

The questionnaire was organized in a first section gathering sociodemographic data regarding age, educational and marital status, job type, visual disability classification (only for visually impaired women), and information about actual and previous physical activity/sport practice. The subsequent section included multiple choice questions investigating the individual attitude toward sport and, in particular, personal reasons underlying sport practice, BXC engagement, and adhesion to the “red diamonds” event. The final part of this section included questions inquiring issues specifically regarding female sport practice and management, as well as the perceived psychological pressure. The last survey section comprised the 18-item Psychological Well-Being (PWB-18) scale and the 12-item Short Form (SF-12) Questionnaire to assess six components of wellbeing (i.e., autonomy, environmental mastery, personal growth, positive relations with others, purpose in life, and self-acceptance), and quality of life, respectively (46). In addition, it also included the Rosenberg Self-Esteem Scale (RSES), a 10-item qualitative tool investigating global self-esteem using multiple choice questions scored through a direct or reverse 0- to 4-point Likert scale (47). The 35-item version of the Dresden Body Image Questionnaire (DKB-35) was administered to assess body satisfaction through 35 items attributable to five subscales inquiring body acceptance, vitality, self-aggrandizement, physical contact, and sexual fulfillment. All the subscales include both directly and reversely scored items, rated through a 1- to 5-point Likert scale (48). Concerning both RSES and DKB-35, a higher score corresponds to a greater self-esteem and a mastered overall body image level, respectively. Finally, body shape awareness and beauty/attractiveness ideals were investigated using, for the first time in visually impaired women, a visual and descriptive tool habitually applied in the armochromy body shaping analysis (49). Specifically, this qualitative tool provides five coded body shape options (i.e., apple, pear, hourglass, oval, and rectangle) and requires choosing the one that better represents own body silhouette, the personally desired one, as well as the subjective beauty ideal one, both in general and supposed to be more attractive for men. Regarding the sighted participants, options were also available as a picture, in addition to the corresponding written description accessible by visually impaired subjects. Adding a description of the available options, written following the armochromy-specific parameters, allowed us to apply such a tool even to our visually impaired target, thus collecting totally comparable data from all participants.

### 2.3 Statistical analysis

Data were entered and stored using Microsoft Office Excel. All data are represented as mean ± standard deviation (SD), or the number or percentage of study subjects. SPSS version 25.0 (Statistical Package for the Social Sciences, Chicago, IL, USA) was used for statistical analyses. Differences between two groups were analyzed by unpaired Student's *t*-test or chi-square test, as appropriate, after verifying the normality of data with a Kolmogorov–Smirnov test. Values of *p* <0.05 were considered statistically significant.

## 3 Results

A total sample of 33 women involved in the “red diamonds” match, comprising 13 visually impaired BXC players [mean age: 32.84 ± 12.05 years; 7 (53.8%) blind and 6 (46.2%) severely sight-impaired subjects], of whom 10 (76.9%) were congenitally visually impaired while 3 (23.1%) had acquired vision loss, and 20 sighted on-field subjects (mean age: 47.15 ± 12.31 years) voluntarily joined this study by responding the self-administered online survey. The collected data regarding sociodemographic characteristics (i.e., age, educational level, employment status, and relationship status) revealed no significant difference between visually impaired and sighted women groups (Table 1). A statistically significant difference in mean age was only observed between the two groups (*p* < 0.05).

**Table 1:** Sociodemographic data of study participants.

Variables	BXC players *n* = 13	Sighted on-field subjects *n* = 20
Age (years), mean ± SD	32.84 ± 12.05	47.15 ± 12.31
Educational level, *n* (%)		
Middle school degree	2 (15.4)	4 (20)
High school degree	9 (69.2)	7 (35)
University	2 (15.4)	9 (45)
Employment status, *n* (%)		
Employee	6 (46.1)	7 (35)
Student	2 (15.4)	3 (15)
Retiree	1 (7.7)	2 (10)
Unemployed	2 (15.4)	1 (5)
Other	2 (15.4)	7 (35)
Relationship status, *n* (%)		
In a relationship	6 (46.1)	12 (60)
Single	7 (53.9)	8 (40)n

Regarding the evaluation of psychological wellbeing and perceived quality of life assessed through PWB-18 scale and SF-12 questionnaire, respectively, mean score results are reported in Table 2.

**Table 2:** Mean scores of the Psychological Well-Being scale and Quality of Life questionnaire in visually impaired baseball players compared with sighted on-field subjects.

Variables	BXC players mean (SD)	Sighted on-field subjects mean (SD)	*p*-value^a^
Psychological Well-Being 18			
Autonomy	10.69 (3.31)	11.70 (2.88)	NS
Environmental mastery	9.23 (2.38)	11.50 (2.21)	0.009
Personal growth	12.23 (3.11)	13.45 (1.19)	NS
Positive relations with others	9.07 (3.59)	9.35 (2.36)	NS
Purpose in life	9.15 (3.18)	11.00 (2.79)	NS
Self-acceptance	10.07 (2.62)	12.40 (2.37)	0.013
Total score	60.46 (12.38)	69.40 (8.14)	0.018
Short Form-12			
Physical	50.31 (10.57)	53.24 (5.60)	NS
Mental	50.11 (11.61)	47.69 (11.00)	NS

NS, non-significant.

^a^ Student's *t*-test for unpaired data.

The comparison between visually impaired and sighted women showed statistically significant differences in the environmental mastery and self-acceptance dimensions of the PWB-18 scale, as well as in the PWB-18 total score, in favor of sighted individuals (Table 2). Conversely, no significant differences in the SF-12 questionnaire score were found between visually impaired baseball players and sighted on-field subjects, though a slightly higher mental index could be observed in the first group (Table 2).

Table 3 displays the comparison of score results concerning body image perception/acceptance and self-esteem level assessed through the DKB-35 questionnaire and the RSES, respectively, between the two groups. Although visually impaired women showed slightly lower scores, both instruments did not reveal any statistically significant difference.

**Table 3:** Mean scores of body image questionnaire and self-esteem scale in visually impaired baseball players compared with sighted on-field subjects.

Variables	BXC players mean (SD)	Sighted on-field subjects mean (SD)	*p*-value^a^
Dresden body image			
Self-aggrandizement	16.53 (5.31)	17.55 (3.79)	NS
Vitality	28.07 (6.00)	28.00 (5.01)	NS
Body acceptance	21.69 (8.23)	23.40 (4.18)	NS
Physical contact	18.53 (4.23)	19.35 (3.92)	NS
Sexual fulfillment	19.76 (6.48)	18.06 (3.83)	NS
Total score	104.61 (23.11)	112.60 (15.18)	NS
Rosenberg self-esteem scale	18.61 (5.69)	21.95 (4.85)	NS

NS, non-significant.

^a^ Student's *t*-test for unpaired data.

Detailing data about body shape perception and the associated beauty ideals, the majority of the entire sample identified the hourglass shape as the most attractive and desirable one [8 out of 13 (61.54%) visually impaired women and 16 out of 20 (80%) sighted women], though just a minority stated that their own bodies conform to it [3 out of 13 (23.8%) visually impaired women and 7 out of 20 (35%) sighted women].

With specific reference to the survey section investigating the sport sociological dimension, three macro inquiring focuses could be identified, the outcomes of which are briefly summarized as follows. The first focus concerned the motivations underlying sport practice/involvement, both in general and BXC related. Responses provided by the study participants revealed a consensus view of sport, particularly BXC, as a tool for social comparison, sharing, and integration. Such results, presented in Supplementary Table S1, are mainly evidenced by the option “*Desire to meet new people and feel part of a group*” chosen by 53.8% of BXC players and 45% of sighted on-field subjects, as well as the option “*Desire to use sport as an integration tool*” chosen by 38.5% and 80% of the aforementioned subjects, respectively. At the same time, a more instrumental approach aimed at self-improvement in the sighted subjects (data emerging from 50% of respondents having selected “*I think sport helps temper character for other challenges*” and 25% “*Enrich my wealth of experience*”) and a more expressive one aimed at self-actualization in visually impaired players *(*“*Competitive spirit*” selected by 38.5% of respondents, and “*Challenge myself*” and “*Desire to challenge myself physically*” by 46.2% and 38.5%, respectively) were also detected. In addition, a different wellbeing concept also emerged between the two groups, markedly more comparable to physical fitness in the sighted than in the visually impaired individuals. Referring to sighted subjects, such a perspective can be inferred from the similar percentages reached by the “*Keep me in good physical shape*” and “*Improve my sense of well-being*” options that were selected by 40% and 45% of respondents, respectively. Conversely, 46.2% of BXC players selected the “*Keep me in good physical shape*” option but did not relate it to wellbeing, as it was inferable by a lower percentage of respondents (23.1%) who chose the corresponding option. Finally, concerning the reasons behind the participation in the “red diamonds” match, both groups asserted that they had been mainly driven by the social value and the visibility opportunity for female sport characterizing the event *(*“*Opportunity to spread an important social message through sport*” and “*Contribute giving visibility to female sport*” options selected by 53.8% and 46.2% of visually impaired BXC players and 75% and 60% of sighted women, respectively). Such motivations were accompanied by the curiosity to experience a women-only version of BXC for sighted subjects (35% of respondents) and by the socialization opportunity for the visually impaired ones *(*“*Desire to challenge myself in a different team group and sport context*” chosen by 38.5% of respondents). The second focus, which inquired about the relationship with the corporeal dimension specifically framed in a sport context, revealed a substantial agreement in perceiving the body as a precious asset to be preserved with the utmost care by both groups (option chosen by 76.9% of BXC players and 60% of sighted on-field subjects). Only the sighted participants also attributed to it a connotation of personal integrity element and environment interaction medium (75% and 55% of respondents, respectively). The third and last investigative focus was on the eventual perception of prejudices and psychological violence in the specific area of female sport. Both groups agreed that no due importance is generally attributed to such a field (61.5% of BXC players and 70% of sighted subjects), as well as that rooted prejudices about the unsuitability of certain sport disciplines for women practice still persist (38.5% of BXC players strongly agreed and 55% of sighted subjects moderately agreed with it) and that female athletes are still portrayed more in terms of esthetics and seductiveness than of their actual athletic skills when compared to the male counterparts (38.5% of BXC players and 45% of sighted subjects moderately agreed with it). Additional biases addressing women sport practice and a devious motherhood boycott within competitive sport were recorded only by the sighted component of the sample (35% strongly agreed and 50% moderately agreed with it). On the request to suggest possible improving/counteracting actions, the visually impaired subjects indicated the need to approach and manage female sport not as a gender-adapted version related to men but as a reality having specific peculiarities (76.9% of respondents). Simultaneously, they also highlighted the necessity of applying equal economic treatment to male and female athletes (53.8% of respondents). In the same perspective, sighted individuals indicated the need for a greater female presence within the management and technical staff of sport societies and federations (70% of respondents). Finally, concerning the possible existence of psychological violence in this specific field, most of the whole women sample stated to be unable to answer (53.8% of visually impaired respondents and 50% of sighted ones). Among the subjects, both sighted and visually impaired, who declared to have perceived/experienced such kind of violence/pressure during their sport practice [7 out of 20 sighted subjects (35%) and 3 out of 13 BXC players (23.1%), respectively], the most responsibility was appointed to managers and coaches (3 out of 3 BXC players and 4 out of 7 sighted subjects) while the audience was held highly responsible by the sighted component only (5 out of 7 respondents).

## 4 Discussion

To the best of our knowledge and starting from the previously demonstrated psychophysical benefits of BXC practice on visually impaired people (20, 46), this is the first study to investigate the social potentialities of such adapted sport not only in the aforementioned target population but also in the sighted component officially provided on-field by the BXC regulation (i.e., assistants, catchers, coaches, and referees). This innovative perspective, along with the event inspiring theme, aimed to inquire about disability and gender discrimination issues increasingly emerging nowadays and potentially contrastable through adapted sport promotion. Since the present investigation was conducted also applying a sociological research approach, our findings must be discussed and framed specifically referring to the Western society to which the investigated sample belongs to. Modern society, strongly based on individuality, productivity, high performative standards, and conformity to the esthetic models imposed by media (50–52), has often considered and represented disabled people as without gender. It is well-known that gender plays a key role in cultural stereotypes of power and performance and, consequently, in discrimination perception/experience and social opportunities. Furthermore, gender stereotypes add to disability-related ones, thus making disabled women one of the most vulnerable and marginalized social category (53).

Due to the deep society transformation caused by globalization, uncontrolled technological progress, media increasing power, and urbanization in the last century, educative and social interaction models faced new difficult challenges (54). Nowadays, human interplay increasingly happens through a virtual approach, which often tends to belittle emotional intelligence, empathy, sense of responsibility, and tolerance that should characterize and drive every interaction. Growingly, we are witnessing bullying and discrimination phenomenon especially affecting the most disadvantaged social groups/individuals, hence highlighting the urgent need to find effective counteracting and educative tools (55, 56).

In such a scenario, sport may represent a promising field through which all these critical issues and challenges can be faced safely and positively. Indeed, the practice of sport, above all adapted team disciplines, urges participants to concretely overcome limits, diversity, and stereotypes to reach a shared goal thus promoting cooperation, reciprocal growth, and integration (19, 57, 58).

On these premises, the present study investigated, for the first time, the complex and multidimensional concepts of psychological wellbeing, quality of life, body image, and perceived sport psychological violence in visually impaired and sighted Italian women playing BXC. The research frame was particularly reach of social meaning given that the “red diamonds” event was conceived and designed to spread a strong message against gender violence and disability discrimination through sport practice. A comparison of the self-administered online survey outcomes, compiled both by visually impaired and sighted participants, revealed no significant differences between the two groups in almost all the investigated variables. Such a general result remarked the already well-demonstrated sport potentialities of counteracting disability-related psychophysical deficits and promoting a constructive interaction between able-bodied and disabled individuals (59–61). Specifically detailing psychological wellbeing, evaluated through the PWB-18 scale, statistically significant differences between sight-impaired and sighted women, in favor of the latter, were detected in the environmental mastery and self-acceptance dimensions, as well as in the total score. Since visual disability deeply and negatively impacts interaction with the surrounding environment and daily-life self-efficacy, such results simply reflect the specific impairment-related issues (4). Moreover, given that the visually impaired women adhering to the study were younger than their sighted counterpart, a lack of environmental experience and self-awareness could be ascribed also to the younger age (62). In addition, though not statistically significant, the SF-12 mental index resulted higher in the BXC players than in sighted on-field subjects confirming the findings of our previous study regarding the benefits of BXC habitual practice in visually impaired individuals (46).

Sport generally promotes a balanced relationship with the corporeal dimension, thus educating practitioners to perceive and use it both as a performative and expressive/relational tool (63, 64). The results concerning body image, evaluated through the DKB-35 questionnaire, revealed no statistically significant differences between the two groups. Visually impaired women showed slightly lower scores in all the areas investigated by the questionnaire except for sexual fulfillment, which might be explained more by the younger age of this group than any other peculiarity (65). Overall, all the women involved in the study achieved a medium to high total score with respect to the established 135 maximum value. It is well-known that self-esteem is strongly related to body acceptance, especially for the female gender (66–68), and such an interconnection can be clearly demonstrated by putting in relation data obtained in the RSES and the specific dimension of the DKB-35 questionnaire. As far as RSES is concerned, the whole women sample reported a total score comprised in the 15–25 range of normality, hence remarking the benefits of sport practice on personality and self-esteem development (69–71). Finally, the survey section investigating body shape perception and the related beauty ideals through the armochromy patterns revealed a common vision within all the participants. Specifically, both sighted and sight-impaired groups stated to consider and desire an hourglass-shaped body as the most attractive but, at the same time, to not perceive so their own. This discrepancy between esthetic ideals and self-body perception might simply highlight the well-demonstrated vision-independent media influence on female beauty models (72, 73). Among visually impaired subjects, blind women particularly appreciated this armochromy-inspired tool, first ever applied in such a target population, finding it useful to mentally visualize/imagine their corporeal shape.

To further deepen the knowledge in blind adapted sport through a multidisciplinary perspective, the present research enriched the evaluative approach investigating not only the psychophysical but also the social dimension of this still under-investigated field. Summarizing the sociological survey outcomes, an instrumental approach to sport emerged in the sighted subjects compared to a more expressive one in the visually impaired group. Such diverse attitudes probably depend on different game roles carried out by the two categories of participants, specifically, assistive/supportive for sighted on-field women and performative for visually impaired players. The different game roles probably also influenced the outcomes regarding possible ameliorative proposals for a concrete female sport acknowledgment. In fact, visually impaired BXC players suggested the application of equal economic treatment to female and male athletes (74), while from sighted on-field subjects emerged the necessity of a greater female presence within the management and technical staff of sport clubs and federations (75). Despite these different perspectives, both groups stated to consider sport as an important sharing, comparison, and integration opportunity, therefore highlighting its educative potentialities on individual psychophysical development and community social growth. In line with such personal belief and the mission of the International Olympic and Paralympic Committee, most of the whole women sample adhered to the “red diamonds” event deeply believing that crucial anti-discrimination social messages can be spread through sport (61, 76, 77). Furthermore, they also stated to have joined this distinctive women-only BXC match considering it as an opportunity of giving to female sport its due importance/visibility (78). The majority of the involved women declared to still perceive many rooted prejudices concerning the suitability of particular sports for female practice and to feel being often portrayed by media more through esthetic than athletic features. Peculiarly referring to the visually impaired players, such perception reaffirmed the widespread gender- and disability-related double discrimination of this vulnerable social target (79). Finally, survey questions that investigated the sensitive topic of perceived psychological violence in female sport showed a weak knowledge or experience of the issue since most of the women choose the “I don't know” option. This finding could be attributed to a real no direct experience of the respondents, as well as to a cultural legacy of silence or to the common devious way of performing prevarication and psychological pressure (80, 81). Among the women who declared to have experienced this kind of violence during their sport practice, the most responsibility was held by coaches. The audience was considered responsible only by sighted subjects, and this could be due to the specific BXC rules imposing it to maintain silence during the match to allow players to hear the auxiliary sound input (20). Consequently, since sight-impaired athletes do not have access to visual feedback, they perceive audience presence and reactions only at the end of each attack/defense action while playing the game.

Given the limits of a self-administered qualitative survey, such speculative hypotheses suggest that further studies on larger samples and different disability categories are needed not only to provide specific knowledge in the field of female adapted sport but, above all, to identify effective tools for preventing and counteracting gender and disability-related discrimination (82–84).

In conclusion, based on our survey results revealing no statistically significant differences in almost all the inquired variables between visually impaired and sighted women involved in the “red diamonds” event, the psychophysical and social benefits of adapted sport have been further deepened and remarked. Specifically, it is to underline that the “dual embodiment” and empowerment phenomenon were investigated, for the first time, in a sociological perspective purpose-tailored for the variegated female component of Italian BXC practitioners. Since the “dual embodiment” process through sport is a crucial dimension in terms of empowerment, gaps reduction, and fight against anti-female and anti-disability prejudice (85, 86), we are confident that our research might help spread interest, knowledge, and awareness of the still under-investigated field of gender and disability disparities in sport. Hopefully, it might also suggest cues for further investigations aimed at deepening and designing educative tools and methodologies to promote a concrete society growth against any form of discrimination through the practice of sport.

## Data Availability

The raw data supporting the conclusions of this article will be made available by the authors, without undue reservation.
