# Supplementary material for: Female blind baseball players against gender discrimination: the “red diamonds” experience
Source: Front Sports Act Living. 2024 Apr 25;6:1362664. doi: 10.3389/fspor.2024.1362664 (PMC11079132; doi:10.3389/fspor.2024.1362664)
Supplement: Supplementary file 1 [file Datasheet1.pdf]

Supplementary Material

Supplementary Table S1. Responses, expressed in number and/or percentage, provided by the study participants in the three macro focuses of questionnaire inquiring i) the motivations underlying sport practice/involvement, both in general and BXC related, and the reasons behind participation in the "red diamonds" match, ii) the relationship with the corporeal dimension specifically framed in a sport context, and iii) the perception of prejudices and psychological violence in the specific area of female sport.

**SURVEY SOCIOLOGICAL SECTION**		
**MOTIVATIONS UNDERLYING SPORT PRACTICE**		
**What are your main motivations for playing sports? (Please choose three options only)**	**BXC players n (%)**	**Sighted on-field subjects n (%)**
Competitive spirit	5 (38.5)	3 (15)
Keep me in good physical shape	6 (46.2)	8 (40)
Challenge myself	6 (46.2)	5 (25)
Improve my sense of well-being	3 (23.1)	9 (45)
Share an activity with familiar people	6 (46.2)	4 (20)
Feel more integrated into society	2 (15.4)	1 (5)
Enrich my wealth of experience	1 (7.7)	5 (25)
Therapeutic purposes	0 (0)	3 (15)
Do an activity that puts me in touch with nature	0 (0)	4 (20)
Advice from parents and/or relatives	0 (0)	0 (0)
Recreation and fun	4 (30.8)	3 (15)
Improve my adaptation to the environment	0 (0)	1 (5)
I think sport helps temper character for other challenges	4 (30.8)	10 (50)
**What motivated you most to approach BXC? (Please choose three options only)**		
Passion for outdoor activities	3 (23.1)	6 (30)
Desire to challenge myself physically	5 (38.5)	1 (5)
Health-related motivation	1 (7.7)	0 (0)
Competitive spirit	4 (30.8)	3 (15)
Desire to meet new people and feel part of a group	7 (53.8)	9 (45)
Desire to use sport as an integration tool	5 (38.5)	16 (80)
**What motivated you most to join the Red Diamonds event? (Please choose three options only)**		
Opportunity to experience a women-only version of BXC	1 (7.7)	(35)
Opportunity to spend time among women and make new friends	5 (38.5)	1 (5)
Contribute giving visibility to female sport	6 (46.2)	12 (60)
Opportunity to play a BXC match even during pre-season phase	0 (0)	1 (5)
Desire to challenge myself in a different team group and sport context	5 (38.5)	3 (15)
Opportunity to spread, through sport, an important social message	7 (53.8)	15 (75)
**RELATIONSHIP WITH THE CORPOREAL DIMENSION**		
**What does body represent for you? (Please choose three options only)**	**BXC players n (%)**	**Sighted on-field subjects n (%)**
An integral part of my being	3 (23.1)	15 (75)
An asset that should be taken the utmost care of	10 (76.9)	12 (60)
An element that has great aesthetic significance	2 (15.4)	2 (10)
The medium through which we experience the world	5 (38.5)	11 (55)
A performance machine	2 (15.4)	1 (5)
A term of comparison between oneself and others	0 (0)	2 (10)
The place of good and bad sensations	3 (23.1)	7 (35)
A thing I am satisfied with	3 (23.1)	1 (5)
A thing I wish it were different	2 (15.4)	2 (10)

PERCEIVED PREJUDICES AND PSYCHOLOGICAL VIOLENCE IN FEMALE SPORT		
**Do you think that female sport is given due importance?**	**BXC players n (%)**	**Sighted on-field subjects n (%)**
Yes	2 (15.4)	3 (15)
No	8 (61.5)	14 (70)
I don't know	3 (23.1)	3 (15)
**Do you think there are still negative prejudices toward the idea of women playing sports?**		
Yes	2 (15.4)	14 (70)
No	8 (61.5)	2 (10)
I don't know	3 (23.1)	4 (20)
**What do you think would be most helpful in making female sport practice less complicated? (Please choose three options maximum)**		
More media coverage	3 (23.1)	6 (30)
A communication campaign against prejudice	5 (38.5)	7 (35)
Perceive and approach female sport as a reality in its own and not as a gender-adapted version of male one	10 (76.9)	13 (65)
Receive the equal economic treatment and aid applied to male sport	7 (53.8)	7 (35)
Receive adequate welfare and assistance measures	1 (7.7)	3 (15)
A more adequate regulation for maternity rights	2 (15.4)	2 (10)
A greater female presence within the management, technical and medical staffs, both in sport clubs and federations	4 (30.8)	14 (70)
Increase the experimental gender-mixed practice in all the sport disciplines	4 (30.8)	3 (15)
**Would you please tell us how much you agree with the following statements?**		
**In sport context, women are still overrepresented in terms of aesthetics and seductiveness**		
Strongly agree	4 (30.8)	5 (25)
Moderately agree	5 (38.5)	9 (45)
Slightly agree	3 (23.1)	6 (30)
Not at all agree	1 (7.7)	0 (0)
**There is still a strong prejudice that some sports are not suitable for women practice**		
Strongly agree	5 (38.5)	6 (30)
Moderately agree	5 (38.5)	11 (55)
Slightly agree	2 (15.4)	3 (15)
Not at all agree	1 (7.7)	0 (0)
**Even though deviously, sport clubs still continue boycotting the motherhood of female athletes**		
Strongly agree	2 (15.4)	7 (35)
Moderately agree	4 (30.8)	10 (50)
Slightly agree	6 (46.2)	3 (15)
Not at all agree	1 (7.7)	0 (0)
**Sport organizations place less importance on female sport than on male one**		
Strongly agree	5 (38.5)	9 (45)
Moderately agree	6 (46.2)	7 (35)
Slightly agree	0 (0)	4 (20)
Not at all agree	2 (15.4)	0 (0)
**In your opinion/perception, is there a high degree of psychological violence in female sport?**		
Yes	3 (23.1)	7 (35)
No	3 (23.1)	3 (15)
I don't know	7 (53.8)	10 (50)
**In case you answered "Yes" to the previous question, which subjects does this pressure come from?**		
**From opponents**		
Yes	2	2
No	1	3
Does not answer	0	2

**From coaches or trainers**		
Yes	3	4
No	0	2
Does not answer	0	1
**From club leaders/manager**		
Yes	3	5
No	0	2
Does not answer	0	0
**From the audience**		
Yes	1	5
No	1	1
Does not answer	1	1
**From family members**		
Yes	1	1
No	1	3
Does not answer	1	3
